# Popular extreme sea level metrics can better communicate impacts

**DOI:** 10.1007/s10584-021-03288-6

**Published:** 2022-02-15

**Authors:** D. J. Rasmussen, Scott Kulp, Robert E. Kopp, Michael Oppenheimer, Benjamin H. Strauss

**Affiliations:** 1 Princeton School of Public & International Affairs, Princeton University, Princeton, NJ USA; 2 Climate Central, Princeton, NJ USA; 3 Department of Earth & Planetary Sciences, Rutgers University, New Brunswick, NJ USA; 4 Institute of Earth, Ocean, & Atmospheric Sciences, Rutgers University, New Brunswick, NJ USA; 5 Department of Geosciences, Princeton University, Princeton, NJ USA; 6 High Meadows Institute, Princeton University, Princeton, NJ USA

**Keywords:** Extreme sea level, Assessment reports, IPCC, Sea level rise, Impacts

## Abstract

Estimates of changes in the frequency or height of contemporary extreme sea levels (ESLs) under various climate change scenarios are often used by climate and sea level scientists to help communicate the physical basis for societal concern regarding sea level rise. Changes in ESLs (i.e., the hazard) are often represented using various metrics and indicators that, when anchored to salient impacts on human systems and the natural environment, provide useful information to policy makers, stakeholders, and the general public. While changes in hazards are often anchored to impacts at local scales, aggregate global summary metrics generally lack the context of local exposure and vulnerability that facilitates translating hazards into impacts. Contextualizing changes in hazards is also needed when communicating the timing of when projected ESL frequencies cross critical thresholds, such as the year in which ESLs higher than the design height benchmark of protective infrastructure (e.g., the 100-year water level) are expected to occur within the lifetime of that infrastructure. We present specific examples demonstrating the need for such contextualization using a simple flood exposure model, local sea level rise projections, and population exposure estimates for 414 global cities. We suggest regional and global climate assessment reports integrate global, regional, and local perspectives on coastal risk to address hazard, vulnerability and exposure simultaneously.

**Supplementary Information:**

The online version contains supplementary material available at 10.1007/s10584-021-03288-6.

## Introduction

Extreme sea levels (ESLs) are short-lived (hours to days), exceptionally high local sea-surface heights, usually resulting from coastal storms, waves, or astronomical tides (Gregory et al. 2019). Observational studies show that contemporary ESLs are occurring with increasing frequency, largely as a result of rising local mean sea level due to global warming and other non-climatic local factors (e.g., ground subsidence; Sweet and Park 2014; Dahl et al. 2017; Menéndez and Woodworth 2010). Future changes in ESL frequency pose significant hazards to coastal communities, natural resources, and ecosystem services (Oppenheimer et al. 2019). Potentially deadly and costly floods can occur in unprepared areas if ESLs overtop natural (e.g., dunes, cliffs) or engineered protection structures (e.g., seawalls, bulkheads, levees). Communicating the risks of changing ESLs can build trust between experts and the public, raise awareness, enhance the understanding of risks, develop agreement about policy options, and motivate pre-emptive risk reduction measures (Rowan 1991). In the case of coastal flooding, the latter includes purchasing flood insurance, elevating assets (e.g., regrading or placing homes on stilts), planning long-term land use strategies (e.g., coastal retreat) and implementing hard protection (Oppenheimer et al. 2019; Rasmussen et al. 2021). Projected changes in ESLs are also crucial for the design of new long-lived infrastructure projects, which can impact future exposure (Rasmussen et al. 2020).

Climate and sea level scientists have developed various metrics and indicators to describe changes in the frequency of contemporary ESLs under various climate change scenarios (Hunter 2012; Buchanan et al. 2017; Rasmussen et al. 2018; Frederikse et al. 2020; Vitousek et al. 2017; Taherkhani et al. 2020; Church et al. 2013; Howard and Palmer 2020; Feng et al. 2018; Fox-Kemper et al. 2021; Tebaldi et al. 2021). For example, ESL amplification factors (AFs; also called “factors of increase” or “multiplication factors”) denote the change in the expected frequency of a given contemporary ESL under a given climate change scenario. ESL frequency AFs denote the expected relative increase in the number of threshold exceedances per year, the threshold usually being an arbitrary return level measured at a tide gauge. In addition to appearing in the primary peer-reviewed literature, ESL AFs have been used to communicate changes in ESLs to policy makers, stakeholders, and the general public in climate assessment reports, such as the Intergovernmental Panel on Climate Change’s (IPCC) Sixth Assessment Report (AR6; Fox-Kemper et al. 2021), Special Report on Global Warming of 1.5 ^∘^C (SR1.5; Hoegh-Guldberg et al. 2018), Special Report on Oceans and the Cryosphere (SROCC; Oppenheimer et al. 2019), and the Fourth U.S. National Climate Assessment Report (U.S. NCA; Sweet et al. 2017).

Despite their wide-spread use, ESL AFs have a few notable limitations in communicating impacts at regional and global scales. First, ESL AFs only consider the hazard component of risk, that being the physical heights of water surfaces. They do not consider corresponding levels of exposure (e.g., population, property value, or natural resources), nor do they consider vulnerability. Some human settlements may be protected to a level above the elevation of the ESL in question (i.e., no flood occurs), or there may exist little to no exposure at or below the ESL height (i.e., a flood occurs, but there is no meaningful impact). Locally, ESLs AFs can be anchored to salient impacts on human systems and the natural environment, such as the frequency of overtopping existing flood defenses, roadway flooding, sewer or drainage back-ups, and the depth of the historically experienced high spring tide (often called a “King Tide”; e.g., Sweet et al. 2018). Without this local information, changes in ESL hazard provide no human or ecological context. Second, ESL AFs and other indicators (e.g., changes in return levels) are generally presented for arbitrary years (e.g., 2050, 2100), which does not provide information about the likelihood of when impacts could cross critical thresholds for a particular location. Third, ESL AFs often highlight single return periods (e.g., the 1-in-100-year ESL, or an event comparable to a specific historic occurrence), potentially neglecting return periods that may be more salient for evaluation of risk for a given location.

In the remainder of this essay, we discuss each of these limitations and show that they can be overcome at regional or global scales. In light of the latter, we provide recommendations for communicating changes in ESLs in future climate assessment reports. Throughout, we use a simple flood exposure model to illustrate our points. Our methods are described in the Appendix.

## Contextualize extreme sea level frequency changes at global scales

Unlike local analyses, regional- and global-scale assessments often do not anchor ESL AFs to impacts, leading to uninformative summary statements that provide little insight for risk communication. For example, the IPCC presently makes summary statements about ESL hazards at the global scale that are based on arbitrary water levels and are not anchored to specific events. This de-contextualizes changes in ESLs. More specifically, in Chapter 4 of the SROCC it is stated, “...extreme sea level event estimates as presented in [Section 4.2.3.4.1], clearly show that as a consequence of sea level rise, events which are currently rare (e.g., with an average return period of 100 years), will occur annually or more frequently at most available locations for RCP8.5 by the end of the century (high confidence)” (Oppenheimer et al. 2019), and also in Chapter 9 of AR6, “Due to relative sea level rise, extreme sea level events that occurred once per century in the recent past are projected to occur at least annually at more than half of all tide gauge locations by 2100 (high confidence)” (Fox-Kemper et al. 2021). While these statements may be true for the hazard, it provides no information about the event exposure or consequence, perhaps leaving the reader to infer that (1) such currently rare events will be destructive wherever they occur or (2) more frequent historical recurrence times are of little importance (e.g., tidal flood impacts that compound over time; Moftakhari et al. 2017).

**Fig. 1:**
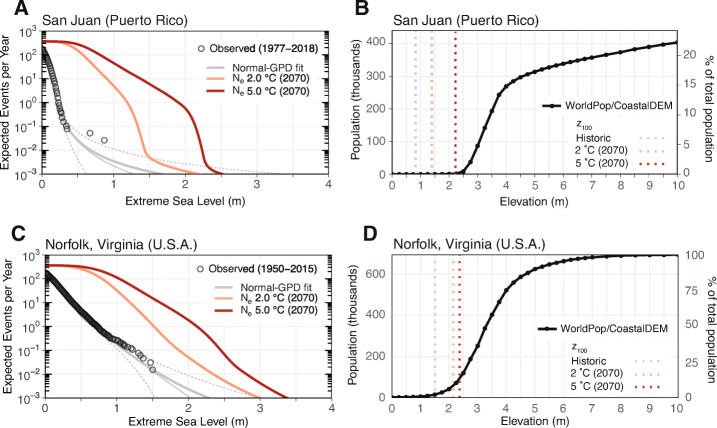
**a** Expected number of contemporary extreme sea level (ESL) events per year as a function of ESL height (meters above local mean higher high water; MHHW) calculated by fitting a Normal-generalized Pareto distribution (GPD) probability mixture model to tide gauge observations (open gray circles) at San Juan (Puerto Rico) for 1991–2009 local mean sea level (thick gray line), expected number of projected ESL events per year as a functions of projected relative sea level change (RSLC) in 2070 under a scenario in which global mean surface air temperature (GSAT) is stabilized in 2100 at + 2 ^∘^C (orange line) and + 5 ^∘^C (red line; GSAT relative to 1850–1900). Thin gray lines are the contemporary ESL return curves for the 5/50/95 percentiles of the GPD parameter uncertainty range (dotted/solid/dotted lines, respectively). **b** A population exposure function that estimates the total population (left y-axis) and percent of total population (right y-axis) currently exposed as a function of ESL height (meters above MHHW) for San Juan (total population: 1.82 million). Filled black circles are population data from the 2010 WorldPop global gridded population database (Tatem 2017) applied to the elevation surfaces of CoastalDEM (Kulp and Strauss 2018). Linear interpolation is used to produce a continuous curve between the WorldPop data (black line). City boundaries are those as defined by Kelso and Patterson (2012) and may differ from actual administrative borders. Populations are assumed to remain constant in time. Denoted is the elevation of the contemporary 100-year ESL (gray), and the expected heights of the 100-year ESL under a + 2 ^∘^C (orange) and + 5 ^∘^C (red) climate scenario. **c** as for A., but at a tide gauge at Sewell’s Point, near Norfolk, Virginia (USA). **d** As for B., but for the Norfolk/Hampton Roads region of Virginia (USA; total population: 695,000)

For some locations, the contemporary 100-year ESL is impactful, but for other locations it is not. Here we illustrate “impact” as the percent of the 2010 total population of a city that resides on lands at or below the elevation of the 100-year ESL (contemporary or projected). For example, by 2070, the frequency of the contemporary 100-year ESL for San Juan (Puerto Rico) is projected to increase from 0.01 events/year to > 31 events/year, on average, under a scenario in which global average surface air temperature (GSAT) stabilizes at + 2 ^∘^C above pre-industrial levels (an ESL AF of $\sim $3100; Table 1; Fig. 1a). However, < 0.1% of the 2010 total population of San Juan, Puerto Rico (< 1,000 out of 1.8 million people) resides on lands at or below the elevation of the contemporary 100-year ESL (Fig. 1b), as estimated using return levels from a local tide gauge and the bathtub flood modeling approach (see Appendix for details). The associated expected relative increase in population below the 100-ESL from projected SLR is $\sim $40% but is still < 1,000 people in absolute terms. Overall, an increase in the frequency of the contemporary 100-year ESL will impact relatively few San Juan residents (assuming constant population). On the other hand, $\sim $2.3% of the total 2010 population of the Norfolk/Hampton Roads region of Virginia, USA ($\sim $16,000 out of 695,000 people), resides on lands below the elevation of the contemporary 100-year ESL, which is expected to occur more than three times per decade by 2070 (ESL AF of $\sim $32; Table 1, Fig. 1d). Despite the ESL frequency AF for Norfolk being almost 100 times less than San Juan, the associated increase in population below the contemporary 100-year ESL is $\sim $3 times larger (Fig. 1c, d; Table 1).

**Table 1:** Table listing both physical and societal extreme sea level (ESL) metrics for select major coastal cities. Given are the heights of the contemporary 100-year ESL return period (meters relative to mean higher high water; expected/5th/95th percentile), the percent of the total population exposed to the expected 100-year ESL, 2070 probabilistic relative sea level change (RSLC) (meters, relative to 1991–2009) from a climate scenario in which global mean surface air temperature (GSAT) is stabilized in 2100 at + 2 ^∘^C (relative to 1850–1900; Bamber et al. 2019), ESL return period amplification factors (AFs) for the 100-year ESL, the population exposure AF, the estimated total population exposed to the projected 100-year ESL (thousands), the same number as a percent of the total population, and the year in which the population exposed to the 100-year ESL doubles. The expected value and the 5/95 percentile of the estimate are given for each. The 5/95 percentile for the contemporary ESL return period considers the uncertainty in the generalized Pareto distribution (GPD) parameters, while the 5/95 percentile for RSLC and AFs reflect the uncertainty from both contributions to local RSLC and from the GPD. The * denotes instances of when the height of the contemporary 100-year ESL occurs more often than the present-day frequency of exceeding MHHW (given for each tide gauge in the supporting information). The mapping of tide gauges to cities is given in the supporting information

100-year ESL event			2070 (2.0 ^∘^C)					
	Contemporary		Physical metrics		Societal metrics			
City	100-year ESL	% Pop	RSLC (m)	ESL frequency AF	Pop exposure	Pop exposed	% Pop exposed	Pop doubling year
(total population in thousands)	(m)	exposed			AF	(thousands)		
Buenos Aires, Argentina (11,980)	2.6 (2.1–3.3)	7.6%	0.4 (0.2–0.7)	3 (2–6)	1.5 (1.2–1.7)	1,328 (1,121–1,526)	11.1% (9.4–12.7%)	2211 (2110–2300)
Copenhagen, Denmark (1,337)	1.1 (1.0–1.1)	1.5%	0.2 (− 0.8–1.1)	991 (0–9677)	1.3 (0.0–3.2)	26 (0–63)	1.9% (< 0.1–4.7%)	2190 (2045–2300)
Dar es Salaam, Tanzania (2,322)	0.7 (0.6–0.7)	1.0%	0.5 (0.2–0.8)	2441 (254–6678)	1.7 (1.1–2.6)	39 (25–59)	1.7% (1.1–2.6%)	2086 (2052–2144)
Hamburg, Germany (1,854)	4.0 (3.6–4.3)	14.9%	0.4 (0.1–0.7)	4 (2–9)	1.1 (1.0–1.2)	301 (285–320)	16.2% (15.3–17.2%)	2300 (2300–2300)
Hong Kong, China (22,232)	1.8 (1.2–2.6)	33.1%	0.4 (0.1–0.8)	5 (1–12)	1.2 (1.1–1.4)	9,015 (7,788–10,131)	40.6% (35.0–45.6%)	2300 (2300–2300)
Honolulu, HI, USA (466)	0.4 (0.3–0.4)	0.5%	0.5 (0.2–0.9)	12385 (942–14455)	4.6 (2.1–8.5)	11 (5–20)	2.3% (1.0–4.2%)	2041 (2025–2065)
London, England (9,878)	0.9 (0.7–1.1)	1.8%	0.4 (0.2–0.7)	61 (4–188)	2.1 (1.4–2.9)	367 (252–515)	3.7% (2.5–5.2%)	2075 (2044–2109)
Manila, Philippines (5,782)	0.8 (0.7–0.9)	36.6%	0.9 (0.6–1.2)	15146 (3018–*)	1.1 (1.1–1.2)	2,339 (2,251–2,443)	40.5% (38.9–42.3%)	2300 (2300–2300)
New Orleans, LA, USA (711)	2.3 (1.2–4.2)	77.7%	1.0 (0.7–1.3)	4 (2–7)	1.2 (1.1–1.2)	643 (623–663)	90.4% (87.6–93.2%)	—
New York, NY, USA (12,520)	1.9 (1.5–2.3)	3.7%	0.6 (0.3–0.9)	11 (2–29)	1.4 (1.2–1.7)	653 (539–799)	5.2% (4.3–6.4%)	2169 (2089–2300)
Norfolk, VA, USA (695)	1.5 (1.1–1.9)	2.3%	0.6 (0.4–1.0)	32 (4–81)	4.0 (2.2–7.3)	64 (35–114)	9.2% (5.1–16.4%)	2042 (2029–2060)
Phuket, Thailand (159)	0.9 (0.8–1.0)	9.0%	0.5 (0.2–0.8)	1723 (37–7875)	1.2 (1.1–1.4)	17 (16–20)	11.0% (9.9–12.5%)	2204 (2104–2300)
Rio de Janeiro, Brazil (9,110)	0.9 (0.8–1.1)	0.3%	0.5 (0.2–0.8)	1061 (8–5808)	1.8 (1.3–2.5)	58 (41–79)	0.6% (0.5–0.9%)	2092 (2055–2165)
San Diego, CA, USA (2,323)	0.7 (0.7–0.7)	0.2%	0.5 (0.2–0.8)	4726 (298–15431)	3.0 (1.6–5.7)	13 (7–25)	0.6% (0.3–1.1%)	2060 (2037–2093)
San Juan, Puerto Rico (1,821)	0.7 (0.5–1.1)	0.0%	0.5 (0.2–0.8)	3130 (4–*)	1.4 (1.1–1.9)	< 1 (< 1–< 1)	< 0.1% (< 0.1–< 0.1%)	2134 (2074–2300)
Shenzhen, China (12,518)	1.8 (1.2–2.6)	17.5%	0.4 (0.1–0.8)	5 (1–12)	1.2 (1.1–1.3)	2,649 (2,314–2,938)	21.2% (18.5–23.5%)	2300 (2300–2300)
Sydney, Australia (3,483)	0.7 (0.7–0.7)	0.2%	0.4 (0.2–0.8)	3213 (60–16480)	1.2 (1.1–1.3)	9 (9–11)	0.3% (0.2–0.3%)	2177 (2090–2300)
Tokyo, Japan (25,339)	1.5 (1.0–2.1)	5.5%	0.4 (0.1–0.7)	8 (1–18)	1.9 (1.1–3.0)	2,651 (1,610–4,278)	10.5% (6.4–16.9%)	2105 (2048–2300)
Vancouver, Canada (1,810)	1.3 (1.1–1.6)	11.8%	0.2 (0.0–0.5)	28 (1–94)	1.0 (1.0–1.0)	218 (214–223)	12.0% (11.8–12.3%)	2300 (2300–2300)

**Fig. 2:**
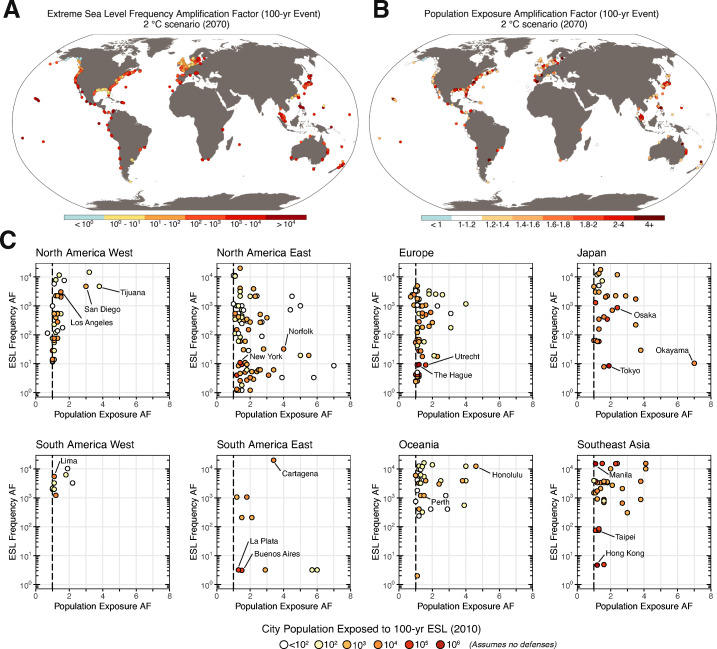
**A** Extreme sea level (ESL) frequency amplification factors (AFs) for cities for 2070 under a climate scenario where the global mean surface air temperature is stabilized in 2100 at + 2 ^∘^C (relative to 1850–1900). **B** As for **A**, but for population exposure AFs. Populations are assumed to remain constant in time. A population exposure AF of 1 indicates no change in exposure. **C** ESL frequency AFs plotted against population exposure AFs for the 100-year ESL for 2070 for the same climate scenario as the maps. The 2010 population exposed to the contemporary 100-year ESL is indicated for each city. A list of the cities in each defined region is given in the supporting data files. Note that some cities may not appear in the scatter plots if (1) contemporary and projected population exposure to flood is zero, (2) the contemporary population exposure to flood is zero but projected exposure is non-zero (i.e., a population exposure AF of infinity), or (3) the population exposure AF is greater than two standard deviations from the mean of each region

To illustrate this discrepancy globally, we consider both the expected change in the frequency of the contemporary 100-year ESL at tide gauges matched to 414 global cities (ESL frequency AF; Fig. 2a) and the expected relative change in number of people in each city currently residing on lands below the elevation of the contemporary 100-year ESL (the “population exposure AF”; Fig. 2b). We then group the cities by geographic region to look for regional patterns (Fig. 2c). Relationships between these metrics vary by city, in part due to differences in projected relative sea level change (RSLC; Gregory et al. 2019) and the shape of both the ESL return curves and the population versus elevation profiles (e.g., Fig. 1b, d). Across all regions, there is no strong relationship between changes in the frequency of the 100-year ESL and changes in population exposure to the 100-year event. Thus, ESL frequency AFs are, by themselves, poor proxies for impact, shown here in terms of total population below the elevation of the 100-year ESL event. Rather than a summary statement that considers only physical changes in ESL frequencies, one could—using this analysis—construct a statement that considers aggregate population exposure changes, “projected 2070 RSLC under an end-of-century + 2 ^∘^C GSAT stabilization scenario (relative to pre-industrial) is expected to at least double the 2010 population residing on lands below the 100-year ESL elevation for 25% of the 414 coastal cities assessed, growing to 37% under an end-of-century + 5 ^∘^C GSAT scenario.”

## Communicate uncertainty in the timing of crossing impact-relevant thresholds

Extreme sea level AFs are often presented for an arbitrary future year in which the projection uncertainty in RSLC is only considered for that particular year (e.g., 2050, 2070, 2100). However, also of interest is analyzing projection uncertainty in the rates of RSLC and how it impacts the timing of reaching ESL frequency benchmarks, such as the first year in which the contemporary 100-year ESL becomes the 1-year ESL. Just as with assessing ESL AFs for particular years, impactful communication of the timing of such benchmarks should be tied to relevant societal thresholds, rather than arbitrary water levels. Examples include the year in which ESLs higher than the design height benchmark of protective infrastructure (e.g., the 100-year water level) are expected to occur within the lifetime of that infrastructure (Rasmussen et al. 2020), or the population exposure associated with a given amount of RSLC. We use our simple ESL population exposure model to illustrate the latter.

**Fig. 3:**
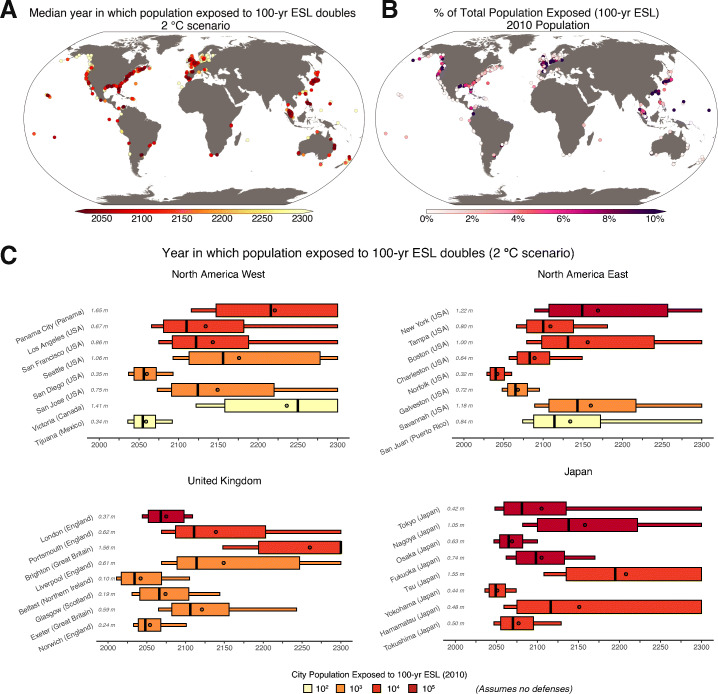
**A** Median projected year in which local relative sea level change (RSLC) doubles the population exposure to the contemporary 100-year extreme sea level (ESL) event (i.e., a population exposure amplification factor of 2; analysis assumes constant population) under a scenario in which global mean surface air temperature in 2100 is stabilized at + 2 ^∘^C (relative to 1850–1900). **B** Percent of the total city population exposed to the contemporary 100-year ESL (assumes 2010 population). **C** As for A, but highlighting select cities to show the RSLC uncertainty as a box plot. The thinner boxes cover the 5/95 percentile of RSLC uncertainty while the thicker boxes cover the 17/83 percentile. Black lines denote the 50th percentile and black dots denote the expected year. The RSLC amounts associated with each population exposure AF threshold are given in light gray (relative to 2000). The color of each box indicates the 2010 population exposure to the 100-year ESL (assumes no flood defenses)

The uncertainty in the timing of a doubling of the population exposed to the 100-year ESL is illustrated for several global cities under a + 2 ^∘^C scenario in Fig. 3 (all cities are given in the Supporting Data). Only uncertainty in the rates of RSLC are accounted for. For the Norfolk/Hampton Roads region, a doubling of the population exposure to the 100-year ESL ($\sim $16,000 people, corresponding to a RSLC of 0.32 m) is likely (17–83% probability) to occur between 2033 and 2051 under a + 2 ^∘^C global mean temperature stabilization scenario (USA; Fig. 3c). For San Juan (Puerto Rico), a doubling of the population exposure to the 100-year ESL is likely (17–83% probability) to occur much later, between 2088 and 2172 (Fig. 3c).

While these analyses are local, they can be aggregated to regional or global scales to better inform aggregate statements about uncertainty in the timing of changes in ESLs. For example, using our analysis, a summary statement could be constructed that communicates uncertainty in the timing of population exposure doubling, “projected 2070 RSLC under an end-of-century + 2 ^∘^C GSAT stabilization scenario (relative to pre-industrial) is expected to at least double the 2010 population residing on lands at or below the elevation of the 100-year ESL before 2100 at 27% of the 414 coastal cities assessed. This fraction grows to 54% under an end-of-century + 5 ^∘^C GSAT scenario.” We note that considering population exposure produces drastically different summary statements from those given in SROCC that only consider the hazard, for example stating that “most” locations will experience the contemporary 100-year ESL annually by 2100 (see Section 2). Furthermore, as opposed to “many” localities experiencing the contemporary 100-year ESL annually by 2050 (as stated in SROCC), we find that under a + 2 ^∘^C GSAT scenario, < 7% of the 414 cities from this study are expected to experience a doubling of the population residing on lands at or below the elevation of the 100-year ESL before 2050 ($\sim $12% under a + 5 ^∘^C GSAT scenario). The focus on a doubling of population exposure is an arbitrary example; other societal thresholds could be explored.

## Further suggestions for ways forward

Extreme sea level (ESL) frequency amplification factors (AFs) are easy-to-calculate metrics that can help communicate flood and sea level rise risks when anchored to salient local events, such as the flooding of roadways, property, and other infrastructure (e.g., Sweet et al. 2018). However, stripped of their local context, ESL AFs only measure changes in arbitrary water levels at tide gauges that do not meaningfully aggregate to regional and global scales. In this essay, we have provided suggestions to improve ESL impact messaging in a regional- or global-scale assessment. We make some further remarks here as well as give suggestions for new research directions.

### The IPCC and other assessment reports should leverage exposure and vulnerability datasets

To address the issue of contextualizing local ESL impacts at the global scale, we recommend the IPCC and other climate assessment reports integrate local exposure and vulnerability datasets that have global coverage into their quantitative analyses. Doing so will also facilitate constructing more definitive summary statements regarding ESL impacts. For example, the IPCC’s SROCC “Summary for Policy Makers” gives a strong, but qualitative statement on projected ESL impacts, “[T]he increasing frequency of high water levels can have severe impacts in many locations depending on exposure” (IPCC 2019). Future IPCC assessments should consider coastal flood risk assessment approaches that consider local hazard, exposure, and vulnerability on global scales (e.g., Hallegatte et al. 2013; Abadie et al. 2017; Hanson et al. 2011).

In this essay we have suggested alternative and additional summary statements that could be constructed from population exposure datasets to add more context to projected changes in ESL frequencies (Sections 2 and 3). These are given as illustrative examples only. Employing sophisticated flood inundation modeling (e.g., Bates et al. 2021) and considering plausible socioeconomic shifts that affect exposure (e.g., population change from the Shared Socioeconomic Pathways or SSPs; O’Neill et al. 2014) could be used to construct similar statements. Because impacts can vary by return period, summary statements should also be made for both more frequent and rarer ESLs (e.g., the 10-year and 500-year ESLs, respectively).

While the inclusion of vulnerability and exposure data is possible for assessment reports that are designed to integrate changes in hazards with impacts (e.g., IPCC’s SROCC, SR1.5, and SREX, the U.S. NCA), it is likely to be challenging (if not impossible) to implement in the IPCC’s main assessment reports (e.g., AR6). This is because physical projections are separated from societal impacts by design, the former appearing in the Working Group 1 (WGI) report, ahead of societal impacts that are released roughly 6 months later in Working Group 2 (WGII). Because of this separation, changes in hazards are inherently provided without context until the WGII report is released. In the interim period between these two reports, scientists, the media, and the public are forced to make subjective judgements about the impacts of these hazard changes without their explicit quantification. Potential solutions include tighter integration among working groups in future assessment cycles (e.g., through the assignment of WGI or WGII advisors to WGII and WGI, respectively) or using Special Reports more often, the chapters of which are often more integrated than of those from the main assessment reports (e.g., Chapter 4 from SROCC).

### Create and maintain new publicly available exposure and vulnerability datasets

New and existing datasets of terrain and flood protection elevation can help meet the needs of global assessment reports that seek to contextualize hazards. However, if there are gaps in the literature regarding these data, assessment reports are unlikely to fill the need on their own. Some existing datasets could help. The continually updated Dynamic and Interactive Vulnerability Assessment database (DIVA) is a popular source of vulnerability and exposure data for global-scale coastal flood risk assessments (Vafeidis et al. 2008; Hinkel et al. 2014; Brown et al. 2016; Kirezci et al. 2020; Jevrejeva et al. 2018; Muis et al. 2016; Wolff et al. 2016). This includes socioeconomic data such as capital stock, tourism, and adaptation costs as well as ecological information such as coastal land type (e.g., wetlands, mangroves, beach) and erosion rates (Hinkel and Klein 2009). However, most DIVA studies are economically oriented (e.g., appraising various adaptation approaches using benefit-cost analyses) and consider country- or regional-level entities (the notable exception is ; Hallegatte et al. 2013). City-level information is arguably more relevant for informing decisions in countries that divide political power between local, state/provincial, and national levels (Den Uyl and Russel 2018; Glicksman 2010; Peterson 1981). Furthermore, to our knowledge, DIVA is not publicly available. Therefore, it cannot be used for new integrative analyses done by the IPCC (see the FAIR data principles, Wilkinson et al. 2016; Juckes et al. 2020).

Large uncertainties in global exposure assessments are associated with the accuracy of digital elevation models (DEMs). New near-global-scale DEMs have provided increased accuracy for population exposure assessments (e.g., CoastalDEM, MERIT, and NASADEM; Kulp and Strauss 2019; Yamazaki et al. 2017). In the USA, where high accuracy data derived from airborne lidar exists for which to validate these products, CoastalDEM’s reported vertical error as measured by the root-mean squared error (RMSE) is 2.4 m, with considerable spatial variability. Given the importance of DEMs in flood risk assessment (McClean et al. 2020), further DEM accuracy improvements for these products are needed (Hinkel et al. 2021; Gesch 2018). CoastalDEM,^1^ MERIT and NASADEM are publicly available.

Exposure is not always a good proxy for impacts, particularly in densely built environments where flood protection plays a significant role. Several populations living in low-lying areas around the world (e.g., deltaic regions) are protected by flood protection such as levees, seawalls, and deliberately raised structures (e.g., buildings on stilts; Scussolini et al. 2016; Nicholls et al. 2019). Just like previous flood exposure studies (Neumann et al. 2015; Hanson et al. 2011; Kulp and Strauss 2019; McGranahan et al. 2007; Jongman et al. 2012; Lichter et al. 2011), our exposure estimates do not account for these protection tactics because they can be overtopped and breached. While this omission is common practice in exposure assessment (McClean et al. 2020), it is not always apparent to policy makers, stakeholders, and decision-makers who must interpret such information. In some cases, it has caused confusion from a communications standpoint (e.g., Mussen 2021). While some efforts have been made,^2^ a spatially explicit global database with flood protection footprints, elevations/protection levels, and failure rates remains elusive (Hinkel et al. 2021).

Lastly, while population exposure is highlighted in this essay as a viable metric to communicate ESL impacts, all ESL metrics have limitations in terms of what impacts they communicate. For example, population exposure metrics that are a percent of the total city population ignore absolute numbers of people. Population vulnerability should be considered, including accounting for those who are least able to evacuate a flood event based on age, disability, poverty, and other cause of immobility (e.g., Hurricane Katrina, Eisenman et al. 2007). Other metrics with policy-relevance include projected increases in disaster aid and flood insurance claims, the credit worthiness of municipalities, and metrics that estimate when recovery from major floods begins to be cut short by new events (e.g., Otto et al. 2021). For example, despite Hurricane Sandy having occurred over eight years ago, the New York Metropolitan Transit Authority just completed repairing the damage suffered by New York City’s subway system (Mass Transit Magazine 2021). New research and publicly available datasets are needed in order to highlight these impacts. Advancements could be made through collaborations among researchers in climate adaptation, disaster risk management, and other relevant fields. Ultimately, the choice of which indicators and thresholds to highlight is subjective and depends on what stakeholders, the public, and policy makers view as being the most relevant for their needs.

## Supplementary Information

ESM 1 (MB 2.39)

## Footnotes

1 The 90-m resolution version only

2 Hallegatte et al. (2013) give upper and lower estimates of flood protection for over 100 major cities around the world based on surveyed responses from local experts. But this list is incomplete, these responses have not been verified, and local protection can also vary within a city. Tiggeloven et al. (2020) use the FLOPROS modeling approach (Scussolini et al. 2016) to estimate flood protection, but these calculations have not been locally verified.
