# Supplementary material for: Popular extreme sea level metrics can better communicate impacts
Source: Clim Change. 2022 Feb 15;170(3-4):30. doi: 10.1007/s10584-021-03288-6 (PMC8847277; doi:10.1007/s10584-021-03288-6)
Supplement: Supplementary file 1 — (MB 2.39) [file 10584_2021_3288_MOESM1_ESM.pdf]

Supplementary Information

S-1 Supporting Information

In this study, we use a simple population exposure model with a new global elevation dataset (Kulp and Strauss, 2018, 2019) to highlight the limitations and consequences of using extreme sea level (ESL) metrics that only consider the physical heights of water levels, specifically ESL AFs (frequency and return level) and ESL hazard allowances. We connect ESLs measured at a global network of tide gauges to present-day population exposure for 414 coastal cities around the world. We project future changes in both the frequency and return levels of historical ESLs and the exposure of current populations under two climate change scenarios (Sec. S-1.2). We note that these are not estimates of future population exposure because we do not make future projections of population change. Additionally, we use a simple, static flood exposure approach that has not been globally validated. These projections are merely intended to highlight the limitations of physical ESL metrics when quantifying coastal flood risk. The performance of our population exposure modeling approach has not been extensively validated and is challenged by a lack of observed flood extents for which to validate our model. Sophisticated flood risk analyses are needed to better understand current and future risk levels.

An overview of the sources of information used to generate population exposure estimates are given in Fig. S-1. First, we estimate the present-day probability of ESLs of various heights at a global network of tide gauges using extreme value theory and a long-term record of hourly sea level observations (Sec. S-1.1). Second, we project changes in both the frequency and height of ESLs using local probabilistic projections of relative sea level change (RSLC)[3] that incorporate ice sheet mass loss estimates from structured expert judgment (Sec. S-1.2). Third, we produce 1-dimensional, city-specific functions of population versus ground elevation to estimate the current population exposure to ESLs (Sec. S-1.3). Fourth, by combining the population exposure damage functions with the future estimates of ESLs, we compute the change in the number of people exposed to various ESLs for each city using population exposure AFs (Sec. S-1.4).

S-1.1 Estimating extreme sea levels

S-1.1.1 Tide gauge data

We use long-term records of hourly or sub-hourly sea level observations from quality controlled tide gauges from the University of Hawaii Sea Level Center[4] and also supplement with other tide gauges from the GESLA2 data set (Woodworth et al, 2016). We limit our use of tide gauge records to only those that have record lengths >30 consecutive years in which each year has >80 percent of observations available. In total, we use 360 unique tide gauges, with median and average record lengths of 48 and ∼54 years, respectively (a list of the tide gauges used is given in the supporting information). We estimate the daily maximum sea level for each day in the tide gauge record with > 80 percent of available observations. We note that this temporal resolution only facilitates the estimation of still-water heights. Tide gauges are often placed inside protected harbors. This generally prevents capturing wave setup effects and swash. Wave setup is more likely to be picked up at tide gauges where there are shallow sloping beaches. Moreover, the time averaging of the observations also filters out contributions from waves, which can be significant contributors to extremes (Melet et al, 2018; Arns et al, 2017). To isolate the variation in extreme sea levels (ESLs), we remove the effect of mean sea level (MSL) change by subtracting the annual MSL from each daily maximum value (i.e., values are de-trended). The de-trended daily maximum tide values are then referenced to local mean higher high water (MHHW; relative to the de-trended mean sea level), defined as the average highest high tide at the tide gauge over a given period (here, either 1993–2012 or the last available 19-year period in the record). The local tidal component is not removed.

[3] Relative sea level change is defined as the change in local mean sea level relative to the sea floor or the underwater surface of the solid Earth (Gregory et al, 2019).

[4] retrieved from: https://uhslc.soest.hawaii.edu, January 2020; (Caldwell et al, 2015)

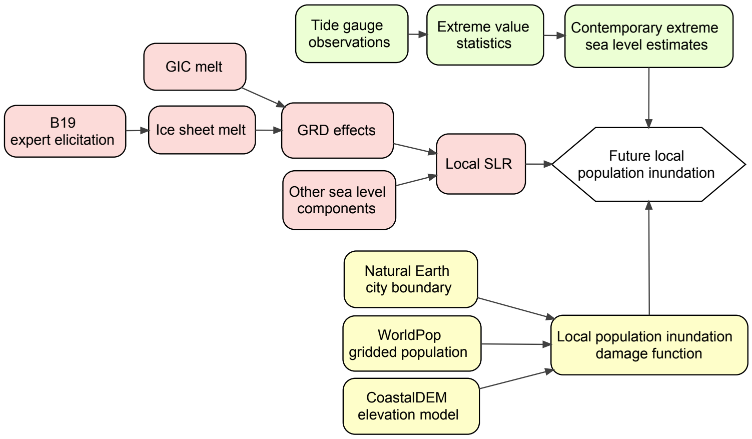

Fig. S-1: Flow of information used in this study to produce projected local population exposure estimates (white hexagon). Green rectangles are for extreme sea level estimation. Red rectangles are for sea-level rise projections. Yellow rectangles are for local population exposure estimates. B19 is Bamber et al (2019); SLR is sea-level rise; GIC are glaciers and ice caps; GRD are gravitational, rotational, and deformational effects; "Other sea level components" includes land water storage, oceanographic processes, and non-climatic background changes, such as glacial-isostatic adjustment.

S-1.1.2 Modeling the probability of rare events with extreme value theory

Extreme value theory is commonly used to produce models that describe unusual or rare behavior (Coles, 2001b; Embrechts et al, 1997). If a modeled fit is considered "good", it can be used to extrapolate the frequency of events of interest, sometimes well beyond the length of the observation record (e.g., determining the height of 100-yr ESL event from a 30-yr record of tide gauge data). Various extreme value distributions and approaches to implement them have been proposed (Coles, 2001b; Embrechts et al, 1997), but in the case of ESL estimation, there currently is not an agreed upon "best approach". Depending on the specific project goals, a particular extreme value modeling strategy may be preferred over another. Following previous studies (Tebaldi et al, 2012; Buchanan et al, 2017; Rasmussen et al, 2018; Frederikse et al, 2020; Wahl et al, 2017), we the extreme values at each tide gauge using a generalized Pareto distribution (GPD; Pickens, 1975; Coles, 2001c,a).

The GPD is a peaks over threshold extreme value modeling approach that describes the probability of a given ESL height conditional on the exceedance of a threshold. The GPD has the advantage over other generalized extreme value (GEV) models for two reasons. First, if the tide gauge record is short, a peaks over threshold approach that uses sub-annual observations/extremes may be preferred over other generalized extreme value (GEV) models that only use annual maxima (i.e., one value per year; Lang et al, 1999; Cunnane, 1973). In other words, more information is retained in the peaks over threshold approach. Using annual maxima can waste data since a year of observations is being represented with a single value. For some locations, more than half of the highest observed ESLs have occurred within the same year (e.g., Boston, U.S.A.; Baranes et al, 2020). Furthermore, if a single annual maxima coincides with a low tidal level, the tidal influence is ignored despite its relevance to estimating ESLs. Second, unlike other GEV distributions (such as the Gumbel; Buchanan et al, 2017), the GPD includes a parameter that allows for the flexibility for the distribution to take on different shapes (shape parameter, ξ, and its value depends on the characteristics of the underlying data), and 3) it can be combined with a Poisson rate parameter to translate ESL exceedance probabilities into expected numbers of annual ESL exceedances (the assumption being that GPD exceedances

are Poisson distributed). The latter may be more intuitive and thus better for communicating the frequency of ESL events.

Following (Coles, 2001a), the cumulative distribution function of the GPD is given by:

$$G(z|u,\alpha,\xi) = \Pr(Z \leq z|Z \geq u) = \begin{cases} 1 - \left(1 + \xi\frac{(z-u)}{\alpha}\right)^{-1/\xi} & \text{for } \xi \neq 0, \\ 1 - \exp\left(-\frac{(z-u)}{\alpha}\right) & \text{for } \xi = 0, \end{cases} \quad \text{(S-1)}$$

with $u \in \mathbb{R}$, $\xi \in \mathbb{R}$, and valid for $\alpha > 0$, $z \geq u$ when $\xi \geq 0$ and $u \geq z \geq u - \alpha/\xi$ when $\xi < 0$. The parameters are as follows, ξ is the shape parameter, α is the scale parameter, and u is the threshold water-level above which return levels are estimated with the GPD.

Selecting the GPD threshold u (sometimes called the "location" parameter–not to be confused with geographic location) is critical element of the peaks over threshold approach as it can have substantial influence on the tail extrapolation. If the threshold is too low, it could bias the estimates because the included values may not be extreme enough. On the other hand, if the threshold is too high, the variance might be too large because too few points are being included in the analysis (Lang et al, 1999). The selection of the threshold can influence the parameter estimation (Coles and Tawn, 1996; Coles and Powell, 1996). GPD thresholds are often chosen either graphically, by looking at a mean excess plot (Embrechts et al, 1997), or by simply setting it to some high percentile of the data (DuMouchel, 1983). The graphical approach is infeasible in this study's case because we are working with several hundred tide gauges. Instead, we set the GPD threshold at the 99th percentile for all sites. The 99th percentile is generally is above the highest seasonal high tide, balances the bias-variance trade-off in the GPD parameter estimation (Tebaldi et al, 2012) and has been found to perform well at global scales (Wahl et al, 2017). Because of the subjective nature of choosing the GPD threshold (Davison and Smith, 1990; Coles and Tawn, 1994), an automated approach remains elusive (Dupuis, 1998; MacDonald et al, 2011).

After the GPD threshold is selected, the daily maximum sea levels above u are de-clustered to satisfy the statistical independence assumption of the GPD. More specifically, the largest daily maximum sea level value within 3 days of each GPD exceedance is used. The remaining GPD parameters are then estimated using maximum likelihood, and their uncertainty is calculated from their estimated covariance matrix. To account for uncertainty in the GPD fit, the estimated covariance matrix is later sampled using a Latin hypercube sampling to produce 1000 normally distributed GPD parameter pairs. Note that the fit of the GPD and the uncertainty bounds may not always well capture the observed exceedances (e.g., San Juan; Fig 1A). While we extrapolate estimates for ESL events up to the frequency of the 1000-yr event, we caution in using any estimate of ESL that exceeds four times the length of the observation record (Pugh and Woodworth, 2014).

The scale, shape, and threshold/location GPD parameters reflect the historical experience with meteorological and oceanographic phenomena that drive ESL events (Pugh and Woodworth, 2014; Coles, 2001c,a; Tebaldi et al, 2012). The shape parameter governs the concavity and upward statistical limit of $G(z)$. Positive shape parameters are indicative of sites exposed to strong tropical cyclones and/or a wide continental shelf that increases storm surge potential (e.g., sites along eastern North America), while negative shape parameters are associated with weaker, infrequent storms, and potentially a narrower continental shelf (e.g., sites in Western North and South America, Fig. S-2A). ESL frequency distributions with $\xi > 0$ are "heavy tailed" and concave up, due to a larger variation in extremes (e.g., existence of tropical and extra-tropical cyclones). Distributions with $\xi < 0$ are "thin tailed", concave down, and have a statistical upper bound on ESLs. The scale parameter α reflects the variability in the distribution of threshold exceedances (i.e., the width of the distribution). The largest scale parameters are found in regions with frequent wintertime extratropical cyclone activity (e.g., the North Sea and Gulf of Alaska; Fig S-2B). The GPD threshold exceedances are shown in Fig. S-2C. The GPD parameters are given in the supporting data files.

S-1.1.3 Mixture normal-extreme value theory probability model

Because the GPD only is defined above the threshold u, an additional distribution is needed to characterize events below u. Following MacDonald et al (2011) and Ghanbari et al (2019), we implement a non-stationary probability mixture model (Eq. S-1.1.3) that simultaneously characterizes the bulk and upper tail of the de-trended daily maximum tide gauge data. The bulk of the data follows a Normal distribution and is fitted as such, however, the upper tail of is not (Fig. S-3. As such, it is considered separately, and a GPD is fit to the

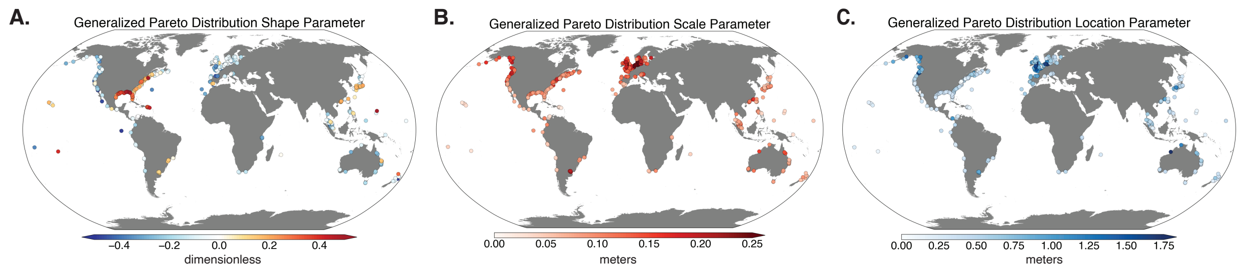

Fig. S-2: **A.** A global map showing the generalized Pareto distribution (GPD) shape parameter (ξ; dimensionless) at cities around the world. **B.** As for A, but for the GPD scale parameter (α; meters above mean higher high water). **C.** As for A, but for the GPD threshold or "location" parameter (u; meters above mean higher high water).

observed extreme values. Studies often ignore the bulk portion of the ESLs distribution, or assume it is well fitted by an arbitrary distribution without empirically testing such fits (e.g., a Gumbel, see Buchanan et al, 2016; Buchanan et al, 2017; Rasmussen et al, 2018, 2020). These are critical assumptions because changes in the most frequent ESLs are the most sensitive to changes in MSL (Buchanan et al, 2017), therefore the increase in the frequency of frequent ESL will be experienced soonest.

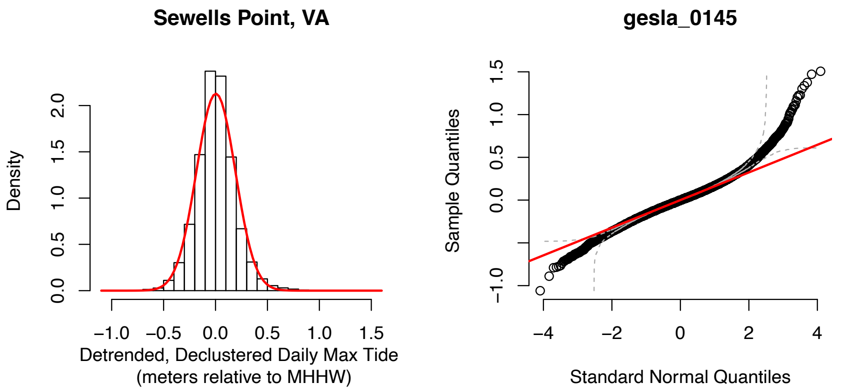

Fig. S-3: **Left:** A histogram of observed maximum daily water levels for Sewell's Point, VA (USA) and a Normal distribution fit (red line). **Right:** As for Left, but a quantile-quantile plot. Note that the bulk of the maximum daily water levels fits a Normal distribution, but the tails do not.

The cumulative distribution function of the mixture model is given by:

$$F(z|\mu,\sigma,u,\alpha,\xi,\varphi) = \begin{cases} (1-\varphi)\,\frac{\mathcal{N}(z|\mu,\sigma)}{\mathcal{N}(u|\mu,\sigma)} & \text{if } z < u, \\ (1-\varphi) + \varphi G(z|u,\alpha,\xi) & \text{if } z \geq u \end{cases} \tag{S-2}$$

where $\mathcal{N}(z|\mu,\sigma)$ and $G(z|u,\alpha,\xi)$ are the Normal and conditional GPD cumulative distribution functions, respectively, and φ is the ratio of de-clustered daily maximum sea level observations that meet or exceed u over the total number of observations. The Normal distribution parameters μ and σ are estimated from the de-trended daily maximum sea level observations, and u is the GPD threshold. An additional benefit of the Normal-GPD probability mixture model (Eq. S-1.1.3) is that it somewhat bypasses the uncertainty in the GPD threshold choice of u through the use of a smooth transition function between the bulk and tail components (MacDonald et al, 2011; Behrens et al, 2004).

To account for local change in mean sea level (described below), the GPD threshold u and the Normal distribution parameter μ linearly shifts. We do not consider future changes in storm frequency (Walsh et al,

2016), intensity (Sobel et al, 2016), or track (Garner et al, 2017), which could all modify these statistical parameters. We also do not consider changes in the tide-surge interaction (Schindelegger et al, 2018; Arns et al, 2017), the surge-sea level interaction (Little et al, 2015), or changes in geomorphology (Familkhalili and Talke, 2016; Talke et al, 2014), all of which may impact future ESL estimates.

For a given tide gauge, the annual expected number of exceedances of height z are given by the Normal-GPD model as follows:

$$N(z) = \begin{cases} n_y \left(1 - \left[\frac{(1-\varphi)}{1+\mathrm{erf}\left(\frac{u-\mu}{\sigma\sqrt{2}}\right)}\left(1+\mathrm{erf}\left(\frac{z-\mu}{\sigma\sqrt{2}}\right)\right)\right]\right) & \text{if } z < u, \\ n_y\varphi\left(1+\frac{\xi(z-u)}{\sigma}\right)^{-\frac{1}{\xi}} & \text{if } z \geq u, \xi \neq 0 \\ n_y\varphi\,\exp(-\frac{z-u}{\sigma}) & \text{if } z \geq u, \xi = 0 \end{cases} \tag{S-3}$$

where n_y is the number of observations per year and erf$(\cdot)$ is the error function. The exceedances are assumed to follow a Poisson process, with a mean of $n_y \times \varphi$.

S-1.2 Relative sea level change projections

Probabilistic, time-varying, local relative sea level change (RSLC) projections for each tide gauge are taken from the component-based study of Kopp et al (2014), except that ice sheet contributions are from the structured expert judgement (SEJ) study of Bamber et al (2019). Projections of RSLC after mid-century are highly dependent on ice sheet melt because of their potential for substantial contributions to global mean sea-level rise (Oppenheimer et al, 2019; Kopp et al, 2019). However, incomplete understanding of the physical processes that govern ice sheet melt inhibits realistic representations in process-based models. In such cases of incomplete scientific understanding, SEJ using calibrated expert responses is one approach for estimating such uncertain quantities (as employed here). Each RSLC probability distribution is conditional on a scenario in which global mean surface air temperature (GSAT) stabilizes in 2100 at either +2 °C (consistent with the Paris Agreement; UNFCCC, 2015) or +5 °C (consistent with unchecked emissions growth; GSAT relative to 1850–1900; Hausfather and Peters, 2020). Samples from each RSLC probability distribution are used to shift the ESL return curves in the direction of the RSLC. Fig. 1A shows the future (2070) ESL return curve for a tide gauge located in San Juan (Puerto Rico). The "kinks" in the return curves appear as a result of the highest samples in the RSLC probability distribution causing the expected ESL frequency calculation to saturate and then subsequently increase the expected number of ESL events. Both the positioning and the presence of the kinks in the return curves are sensitive to the choice of where the upper-tail of the RSLC distribution is truncated (Rasmussen et al, 2020).

The other RSLC component contributions are from ocean thermal expansion, glaciers and ice caps, and land-water storage. General circulation model (GCM) output is used to generate the steric and glacial ice melt sea level components for each global mean surface air temperature (GSAT) stabilization scenario. The +2 °C scenario used the GCM outputs specified for the same GSAT scenario in Rasmussen et al (2018) and the +5 °C scenario used GCM outputs from the representative concentration pathway (RCP) 8.5 from Kopp et al (2014). Also accounted for are regional effects such as ocean dynamics (from GCM output), gravitational and rotational effects of ice mass redistribution, glacial isostatic adjustment, and other local vertical land motion factors (e.g., tectonic uplift, sediment compaction and ground water withdrawal). Probability distributions of local RSLC are produced using 10,000 Latin hypercube samples of each individual sea level component contribution. The probability distributions are truncated at the 99.9th percentile to remove samples that are deemed to be physically implausible (Oppenheimer et al, 2019). As noted by Rasmussen et al (2020), both ESL frequencies and allowances are sensitive to truncation selection. More details and limitations to the RSLC projections are provided elsewhere (Kopp et al, 2014; Bamber et al, 2019; Rasmussen et al, 2020).

S-1.3 Exposure analysis

The vertical population profile of a city can determine the population exposure to a given ESL event. We produce 1-dimensional (vertical) population profiles for 414 global cities using CoastalDEM and population density data from the WorldPop 2010 high resolution (3 arc second) gridded global population data set (Tatem, 2017). In order to simplify our analysis and also isolate the impact of RSLC on population exposure, we assume that population remains fixed in time. Thus, our results are not literal projections of future population exposure—which will depend upon population growth (Hauer et al, 2016; Jongman et al, 2012) and the dynamic response of the population to RSLC (Merkens et al, 2016; Hauer, 2017)—but are instead intended to highlight the impact of ESL events relative to changes in their frequency. A population exposure profile for San Juan (Puerto Rico) is given in Fig. 1B. Exposure profiles for all cities are included in the supporting data.

We map flood extents for each city using the "bathtub" model, a static flood model approach that only considers the vertical elevation of two surfaces, 1) the terrain and 2) a given ESL return level (e.g., 100-yr event) measured at the nearest tide gauge within a 100-km radius of each city center. The return level of interest is spatially extrapolated over the terrain. Static flood approaches have many limitations, including overestimating observed flood extents relative to hydrodynamic models (Breilh et al, 2013; Gallien, 2016; Ramirez et al, 2016; Seenath et al, 2016; Bates et al, 2005; Vousdoukas et al, 2016). However, hydrodynamic modeling is computationally expensive, and producing state-of-the-art projections of population exposure is not the goal of this analysis. Land elevation data are from CoastalDEM, a modified version of NASA's Shuttle Radar Topography Mission (SRTM) digital elevation model (DEM) that uses a neural network trained using lidar-derived elevation data in the U.S. (NOAA, 2020) to reduce SRTM errors (Kulp and Strauss, 2018, 2019; Farr et al, 2007). The SRTM is a near-global DEM commonly used for flood exposure modeling but is known to have large vertical bias (an estimated 3.7 m in coastal areas in the U.S.; Kulp and Strauss, 2018). This is important because coastal flood risk analysis is largely performed within this elevation range and these biases are roughly the same magnitude as projections of future local RSLC over this century (generally < 2 m relative to 2000; Oppenheimer et al, 2019; Gesch, 2018). More details and limitations are provided in Kulp and Strauss (2018, 2019).

Most populations living in low-lying areas around the world (e.g., deltaic regions) are very likely protected by flood defenses such as levees, seawalls, and deliberately raised structures (e.g., buildings on stilts; Scussolini et al, 2016). Following previous flood exposure studies (Neumann et al, 2015; Hanson et al, 2011; Kulp and Strauss, 2019; McGranahan et al, 2007; Jongman et al, 2012; Lichter et al, 2011), our exposure estimates do not account for these defenses because they can be overtopped and breached; only the population in the floodplain is considered. However, protection is assumed to only change the flood hazard and enters our integrated calculations of population exposure that consider all ESL return levels (Sec. S-1.1). To our knowledge, verified, location-specific levels of protection are not available at the global scale[5]. To account for flood defenses that provide a margin of safety, we make multiple arbitrary assumptions regarding the current level of protection for all cities. Specifically, we produce results assuming spatially uniform "no protection" and protection up to the height of the 1- and 10-yr ESL event. These assumptions may greatly differ from reality and could lead to gross over-estimates for cities with existing flood protection that afford a high margin of safety from rare storms, such as London, New Orleans, Tokyo, Shanghai, and most major cities in the Netherlands (Nicholls et al, 2008; Hallegatte et al, 2013; Xian et al, 2018; Scussolini et al, 2016). Despite this, we still include protection assumptions for all cities to 1) limit the inclusion of the lowest elevations which are most prone to vertical errors in the DEM and 2) to highlight the importance of the flood protection assumption and the need for accurate estimates thereof (e.g., Scussolini et al, 2016). The protection assumptions do not impact AFs above the height of the protection level, but can significantly impact integrated metrics that consider all ESLs and impacts, such as the expected annual exposure (EAE; Table S-4)(Kulp and Strauss, 2017).

A vertical adjustment was made to the ESLs to reference the same local mean higher high water (MHHW) datum as CoastalDEM. ESLs use tide gauge data for estimating MHHW, while CoastalDEM uses the glob-

[5] However, Hallegatte et al (2013) and Scussolini et al (2016) give upper and lower estimates of flood protection for over 100 major cities around the world based on surveyed responses from local experts. But these responses have not been verified, and local protection can vary within a city.

ally extensive MSL model MSS CNES-CLS15[6], based on a 1993—2012 record of satellite sea surface height measurements from TOPEX/Poseidon, and MHHW deviations from MSL provided by Mark Merrifield, University of Hawaii, developed using the model TPX08 (Egbert and Erofeeva, 2002). Using the National Oceanic and Atmospheric Administration's (NOAA) VDatum tool (version 3.7; Parker et al, 2003), we convert these measurements to vertically reference EGM96, and we subtract the resulting surface from CoastalDEM to convert its vertical datum to MHHW (adjustments are provided in the supporting data files).

We take a static flood modeling approach by spatially extrapolating return periods of ESLs, measured at tide gauges, onto the surrounding topography. We map one tide gauge to each city. For cities where there is more than one tide gauge within a 100-km radius (e.g., Willet's Point and the Battery in NYC), the tide gauge with the longest record is used (the tide gauges assigned to each city are listed in the supporting data files). While the height of a given ESL return period may vary within a city, in part due to complicated bathymetry and coastlines, Kulp and Strauss (2017) has shown that ESL population exposure results for the U.S. are generally insensitive to tide gauge assignment within a 100-km radius. In any case, static flood approaches do not account for complex local geomorphology, local ocean dynamics, and frictional losses as water moves over different land surfaces (e.g., wetlands, beaches, urban areas). More complex hydrodynamic inundation approaches that model the flow of the ocean onto a variety of land surfaces could be used to more accurately estimate local floods for cities across the globe. However, such an approach is out of the scope of this study as our intention is to merely highlight the limitations of physical ESL metrics. Furthermore, we note that hydrodynamic inundation modeling may not necessarily outperform simpler approaches, especially in active tropical cyclone regions (Hunter et al, 2017; Muis et al, 2016, 2017; Wahl et al, 2017), in part due to poor representation of tide-surge interactions (Arns et al, 2020) and short simulation periods that are less likely to produce rare, extreme events found in multi-decadal tide gauge records. For some locations, the height of the 100-yr ESL event can be under-predicted by up to 3 meters compared to tide gauge-derived estimates (supporting information of Muis et al, 2017).

The WorldPop population data are resampled to align with CoastalDEM raster (for more details, see Kulp and Strauss, 2019), integrated over select elevations (-2 to 13 m above MHHW, either 0.25 or 0.5 m increments), and then tabulated according to the satellite-derived urban footprint for each city from Natural Earth (which may differ from the actual administrative boundary; Kelso and Patterson, 2012). We note that Kulp and Strauss (2019) instead uses LandScan population density at 30 arc seconds. Coastal cities are only included in the analysis if there are populations with in the defined boundary at any elevation < 13 m above local MHHW. Connected components analysis excludes low-elevation inland areas that are not linked to the ocean. Linear interpolation is used between each select elevation to produce a continuous 1-dimensional population exposure profile $D(z)$, where z is the ESL height. We note that z could be a vector to account for spatial differences in ESL height for the same return period within a city and also spatial variation in flood protection.

Exposure analyses are most sensitive to spatially-autocorrelated vertical errors in the DEM at local scales and when assessing population vulnerability at low elevations (e.g., < 0.5 m; Kulp and Strauss, 2019). The higher the elevation that population exposure is being assessed at (e.g., longer return periods, such as the 100-yr event), the less of an impact these errors will have on exposure (Kulp and Strauss, 2019). To assess the impact of elevation errors on population exposure AFs, we use the example of the 100-year ESL event in New York City. Considering lidar topography as ground truth, we find that the EAE AFs are generally insensitive to errors in CoastalDEM; small differences only appear by 2100 for the +5 °C scenario (2.3 vs 2.6; Table S-2). This is despite CoastalDEM underestimating population exposure relative to lidar (connected components analysis not performed for this test; Tables S-1,S-2). We note that CoastalDEM was trained on lidar elevation data in the U.S., so locations outside the U.S. may be more sensitive.

[6] Aviso. MSS CNES-CLS15, https://www.aviso.altimetry.fr/en/data/products/auxiliary-products/mss.html (2015)

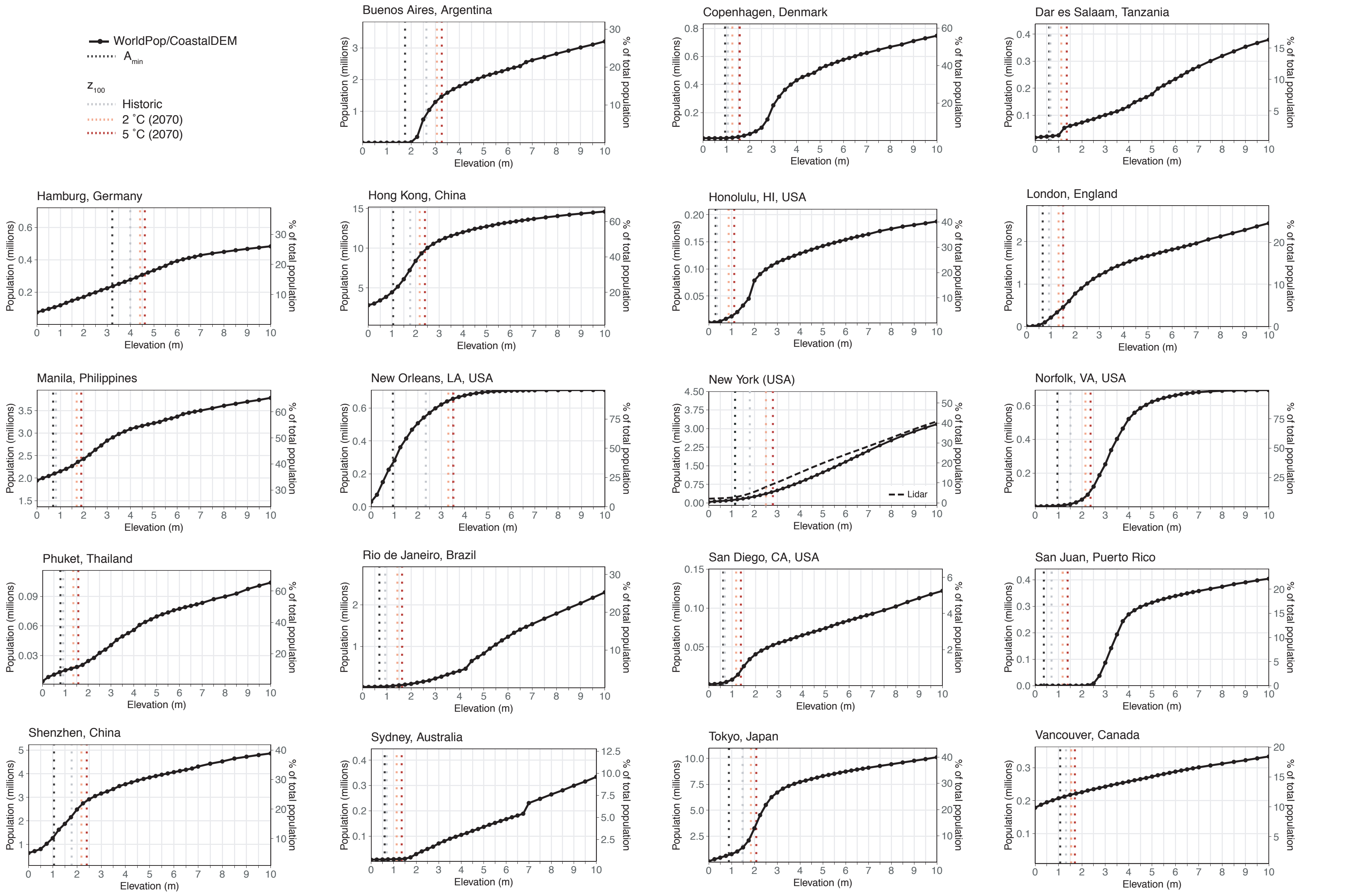

Fig. S-4: Population exposure functions that estimate the total population currently exposed to flooding as a function of ESL height (meters above MHHW) for cities given in Table 1. Filled black circles are population data from the 2010 WorldPop global population database (Tatem, 2017) applied to the elevation surfaces of CoastalDEM (Kulp and Strauss, 2018, 2019). Linear interpolation is used to produce a continuous curve between the WorldPop data (black line). City boundaries are those as defined by Kelso and Patterson (2012) and may differ from actual political boundaries. Populations are assumed to remain constant in time. Denoted is the current level of protection (A_{min}), assumed to be the 10-yr ESL event, the height of the historical 100-yr ESL event (grey), and the expected heights of the 100-yr ESL event under a +2 °C (orange) and +5 °C (red) climate scenario. Also shown for New York City is a population profile generated using a 0.3-m horizontal resolution light detection and ranging (LiDAR)-derived digital elevation model for the City of New York (https://data.cityofnewyork.us/City-Government/1-foot-Digital-Elevation-Model-DEM-/dpc8-z3jc).

S-1.4 Estimating physical and population exposure amplification factors

Following Buchanan et al (2017), the ESL frequency AF for an event of height z^* under uncertain RSLC is $\mathbb{E}[N(z^* - \delta)/N(z^*)]$, where $N(z^* - \delta)$ is the expected number of exceedances of height z^* after considering RSLC δ (Sec. S-1.1). The ESL return level AF is given by $1 + \mathbb{E}[\delta]/z^*$. Here, we extend the ESL return level AF to changes in population exposure. We define the population exposure AF for an event of height z^* under uncertain RSLC as $\mathbb{E}[D(z^* + \delta)/D(z^*)]$, where $D(\cdot)$ is a 1-dimensional vertical population profile of a city (Sec. S-1.3). Note that since $D(\cdot)$ is solely a function of z, the frequency amplification of population exposure events is equivalent to the ESL frequency AFs for the same city. Probability distributions of ESL AFs (frequency and return level) and population exposure AFs are produced for each tide gauge using the RSLC samples for each climate scenario (Sec. S-1.2). Results are then taken from these distributions.

2.0 °C	2010	2070		2100	
	Population (thousands)	Population (thousands)	Pop Exposure AF	Population (thousands)	Pop Exposure AF
Lidar	399 (293-564)	607 (488-745)	1.5 (1.2-1.9)	708 (518-938)	1.8 (1.3-2.4)
CoastalDEM	229 (168-324)	351 (280-440)	1.5 (1.2-1.9)	421 (297-596)	1.8 (1.3-2.6)

Table S-1: Estimated total population exposure and amplification factors for the 100-yr extreme sea level (ESL) event for New York City using 1) a 0.3-m horizontal resolution light detection and ranging (LiDAR)-derived digital elevation model for the City of New York (https://data.cityofnewyork.us/City-Government/1-foot-Digital-Elevation-Model-DEM-/dpc8-z3jc) and 2) CoastalDEM. Future projections assume a climate scenarios in which global mean surface air temperature is stabilized in 2100 at +2 °C (relative to 1850–1900; Bamber et al, 2019).

5.0 °C	2010	2070		2100	
	Population (thousands)	Population (thousands)	Pop Exposure AF	Population (thousands)	Pop Exposure AF
Lidar	399 (293-564)	687 (529-880)	1.7 (1.3-2.2)	917 (617-1,398)	2.3 (1.5-3.5)
CoastalDEM	229 (168-324)	405 (303-548)	1.8 (1.3-2.4)	589 (355-1,010)	2.6 (1.6-4.4)

Table S-2: As for Table S-1, but for a climate scenarios in which global mean surface air temperature is stabilized in 2100 at +5 °C (relative to 1850–1900; Bamber et al, 2019).

100-yr ESL event			2070 (5.0 °C)					
	Historical		Physical metrics			Societal metrics		
City (Total population in thousands)	100-yr ESL (m)	% Pop exposed	RSLC (m)	ESL frequency AF	ESL level AF	Pop exposure AF	Pop exposed (thousands)	% Pop exposed
Buenos Aires, Argentina (11,980)	2.6 (2.1-3.2)	7.5%	0.6 (0.3-1.1)	8 (2-20)	1.2 (1.1-1.4)	1.7 (1.4-1.9)	1,520 (1,241-1,682)	12.7% (10.4-14.0%)
Copenhagen, Denmark (1,337)	1.1 (1.0-1.1)	1.5%	0.5 (0.1-1.0)	841 (10-5575)	1.5 (1.1-1.9)	3.1 (1.1-2.5)	62 (21-51)	4.7% (1.6-3.8%)
Dar es Salaam, Tanzania (2,322)	0.7 (0.6-0.7)	1.0%	0.7 (0.3-1.2)	4720 (620-6678)	2.1 (1.5-2.9)	3.7 (1.1-3.1)	83 (26-70)	3.6% (1.1-3.0%)
Hamburg, Germany (1,854)	4.0 (3.6-4.4)	14.9%	0.6 (0.3-1.1)	9 (2-22)	1.2 (1.1-1.3)	1.2 (1.1-1.2)	321 (293-338)	17.3% (15.8-18.2%)
Hong Kong, China (22,232)	1.8 (1.2-2.6)	32.9%	0.6 (0.2-1.2)	120 (2-155)	1.4 (1.1-1.7)	1.4 (1.2-1.5)	10,334 (8,437-10,847)	46.5% (38.0-48.8%)
Honolulu, HI, USA (466)	0.4 (0.3-0.4)	0.5%	0.8 (0.4-1.3)	14017 (14455-14455)	3.1 (2.0-4.7)	41.2 (3.2-18.4)	96 (7-43)	20.5% (1.6-9.1%)
London, England (9,878)	0.9 (0.7-1.1)	1.8%	0.6 (0.3-1.0)	223 (12-1242)	1.6 (1.3-2.1)	4.2 (1.8-4.1)	750 (310-728)	7.6% (3.1-7.4%)
Manila, Philippines (5,782)	0.8 (0.7-0.9)	36.6%	1.1 (0.7-1.6)	17424 (7870–*)	2.3 (1.9-3.0)	1.3 (1.1-1.2)	2,757 (2,280-2,601)	47.7% (39.4-45.0%)
New Orleans, LA, USA (711)	2.4 (1.2-4.3)	77.9%	1.2 (0.8-1.7)	90 (3-26)	1.5 (1.4-1.7)	1.2 (1.1-1.2)	661 (635-678)	92.9% (89.3-95.3%)
New York, NY, USA (12,520)	1.9 (1.5-2.3)	3.7%	0.8 (0.4-1.3)	228 (3-296)	1.4 (1.2-1.7)	1.8 (1.2-2.1)	837 (578-957)	6.7% (4.6-7.6%)
Norfolk, VA, USA (695)	1.5 (1.1-2.0)	2.3%	0.9 (0.5-1.4)	343 (7-1660)	1.6 (1.3-1.9)	10.7 (2.7-13.9)	169 (42-219)	24.3% (6.1-31.5%)
Phuket, Thailand (159)	0.9 (0.8-1.0)	9.0%	0.7 (0.3-1.2)	6012 (208–*)	1.7 (1.3-2.3)	2.3 (1.1-1.8)	33 (16-26)	20.8% (10.3-16.1%)
Rio de Janeiro, Brazil (9,110)	0.9 (0.8-1.1)	0.3%	0.7 (0.3-1.2)	3951 (30-14619)	1.7 (1.4-2.3)	4.1 (1.5-3.5)	129 (49-110)	1.4% (0.5-1.2%)
San Diego, CA, USA (2,323)	0.7 (0.7-0.7)	0.2%	0.7 (0.3-1.2)	9611 (798-15431)	2.0 (1.5-2.8)	11.0 (2.0-8.6)	48 (9-38)	2.1% (0.4-1.6%)
San Juan, Puerto Rico (1,821)	0.7 (0.5-1.1)	0.0%	0.7 (0.3-1.2)	10597 (11–*)	2.0 (1.5-2.7)	16.3 (1.2-5.3)	2 (<1-1)	0.1% (<0.1- <0.1%)
Shenzhen, China (12,518)	1.8 (1.2-2.6)	17.4%	0.6 (0.2-1.2)	120 (2-155)	1.4 (1.1-1.7)	1.4 (1.1-1.4)	2,993 (2,496-3,131)	23.9% (19.9-25.0%)
Sydney, Australia (3,483)	0.7 (0.6-0.7)	0.2%	0.7 (0.3-1.1)	8942 (374-16480)	2.0 (1.5-2.7)	4.8 (1.1-2.6)	39 (9-21)	1.1% (0.3-0.6%)
Tokyo, Japan (25,339)	1.5 (1.0-2.1)	5.5%	0.6 (0.2-1.2)	435 (2-830)	1.4 (1.1-1.8)	3.8 (1.4-4.2)	5,357 (1,907-5,965)	21.1% (7.5-23.5%)
Vancouver, Canada (1,810)	1.3 (1.1-1.6)	11.8%	0.4 (0.1-0.9)	436 (2-1065)	1.3 (1.0-1.6)	1.1 (1.0-1.1)	232 (215-229)	12.8% (11.9-12.7%)

Table S-3: Table listing both physical and societal extreme sea level (ESL) metrics for select major coastal cities. Given are the heights of the historical 100-yr ESL return period (meters relative to mean higher high water; expected/5th/95th percentile), the percent of the total population exposed to the expected 100-yr ESL event, 2070 probabilistic relative sea-level change (RSLC) (meters, relative to 1991–2009) from a climate scenario in which global mean surface air temperature (GSAT) is stabilized in 2100 at +5 °C (relative to 1850–1900; Bamber et al, 2019), ESL return period amplification factors (AFs) for the 100-yr ESL event, ESL return level AFs for the 100-yr ESL event, the population exposure AF, the estimated total population exposed to the future 100-yr ESL event (thousands), and the same number as a percent of the total population. The expected value and the 5/95 percentile of the estimate are given for each. The 5/95 percentile for the current ESL return period considers the uncertainty in the generalized Pareto distribution (GPD) parameters, while the 5/95 percentile for RSLC and AFs reflect the uncertainty from both contributions to local RSLC and from the GPD. The * denotes instances of when the height of the current 100-yr ESL event occurs more often than the present-day frequency of exceeding MHHW (given for each tide gauge in the supporting information). The mapping of tide gauges to cities is given in the supporting information.

City	Population (millions)	2010 EAE (millions)			2.0 °C								
					2050 EAE AF			2070 EAE AF			2100 EAE AF		
		None	1-yr	10-yr	None	1-yr	10-yr	None	1-yr	10-yr	None	1-yr	10-yr
Shenzhen, China	12.52	1.07	0.70	0.70	1.3	2.0	2.0	1.6	2.5	2.5	2.3	3.5	3.5
Vancouver, Canada	1.81	0.20	0.13	0.13	1.0	1.6	1.6	1.0	1.7	1.7	1.1	1.8	1.8
New York, NY, USA	12.52	0.22	0.15	0.15	1.4	2.1	2.1	1.8	2.6	2.6	2.8	4.1	4.1
London, UK	9.88	0.04	0.03	0.03	3.0	3.6	3.6	5.0	5.9	5.9	9.9	11.7	11.6
Buenos Aires, Argentina	11.98	0.03	0.03	0.03	2.0	2.1	2.1	3.4	3.5	3.5	11.7	12.1	12.2
San Diego, CA, USA	2.32	<0.01	<0.01	<0.01	2.4	4.3	4.3	5.2	9.1	9.1	12.5	21.8	21.8
Rio de Janeiro, Brazil	9.11	0.03	0.02	0.02	1.3	2.2	2.2	2.0	3.3	3.3	4.1	6.7	6.7
Hong Kong, China	22.23	3.99	2.53	2.53	1.2	1.9	1.9	1.4	2.2	2.2	2.1	3.3	3.3
Manila, Philippines	5.78	2.06	1.24	1.24	1.1	1.8	1.8	1.1	1.9	1.9	1.3	2.2	2.2
Copenhagen, Denmark	1.34	0.02	0.01	0.01	2.3	3.8	3.8	4.9	7.8	7.8	15.1	24.4	24.4
Dar es Salaam, Tanzania	2.32	0.02	0.01	0.01	1.2	2.3	2.3	2.2	3.9	3.9	3.3	5.9	5.9
Sydney, Australia	3.48	0.01	<0.01	<0.01	1.1	1.8	1.8	1.2	2.0	2.0	3.2	5.2	5.2
Phuket, Thailand	0.16	0.01	0.01	0.01	2.0	2.3	2.3	2.3	3.4	2.3	3.4	3.4	3.4
Tokyo, Japan	25.34	0.55	0.38	0.38	1.4	2.1	2.1	2.0	2.9	2.9	5.9	8.7	8.7
New Orleans, LA, USA	0.71	0.17	0.13	0.13	2.2	3.0	3.0	2.8	3.7	3.7	3.6	4.7	4.7
Norfolk, VA, USA	0.69	<0.01	<0.01	<0.01	2.0	2.7	6.9	3.4	4.7	13.7	13.9	19.0	55.5
San Juan, Puerto Rico	1.82	<0.01	<0.01	<0.01	2.5	2.6	2.7	3.0	3.1	3.3	16.6	17.3	18.3

Table S-4: expected annual population exposure (EAE; millions) under different assumptions of existing coastal flood protection (no protection and protection against the 1-yr and 10-yr events) and amplification factors (AFs) for the EAE for 2050, 2070, and 2100 under the same coastal flood protection assumptions and under a climate scenario in which global mean surface air temperature is stabilized in 2100 at +2 °C (relative to 1850–1900; Bamber et al, 2019).
